# Case Report of Transverse Myelitis in a Patient Receiving Etanercept for Rheumatoid Arthritis

**DOI:** 10.1155/2013/728371

**Published:** 2013-03-19

**Authors:** Helen Defty, Edward Sames, Teresa Doherty, Rodney Hughes

**Affiliations:** Rheumatology Department, St. Peters Hospital, Guilford Road, Chertsey, Surrey KT16 0PZ, UK

## Abstract

Etanercept is a monoclonal antibody targeted against Tumour Necrosis Factor-alpha (TNF-a) which is an effective treatment for rheumatoid arthritis and is in cases where conventional disease modifying agents such as methotrexate have failed. Neurological complications of treatment have been documented. We describe a case of transverse myelitis occurring in a 48 year-old lady with RA since 1994 who had been receiving etanercept for four years.

## 1. Case Presentation

Mrs. S, a 48-year-old lady with RA of 11 years duration responded well to treatment with etanercept, with an improvement in her Disease Activity Score (DAS) from a pretreatment value of 6.98 in June 2005 to 2.69 within five months.

In December 2008, she began to develop bilateral numbness and tingling in her lower limbs and the ulnar border of her left hand. She also experienced numbness in the perineum and associated bowel urgency. On examination, tone, power, reflexes, and coordination were normal throughout; however she had reduced vibration sense to the waist, joint position sense to the feet, and temperature to the knees bilaterally. Temperature sensation was also reduced in the ulnar fingers of her left hand.

**Figure 1:**
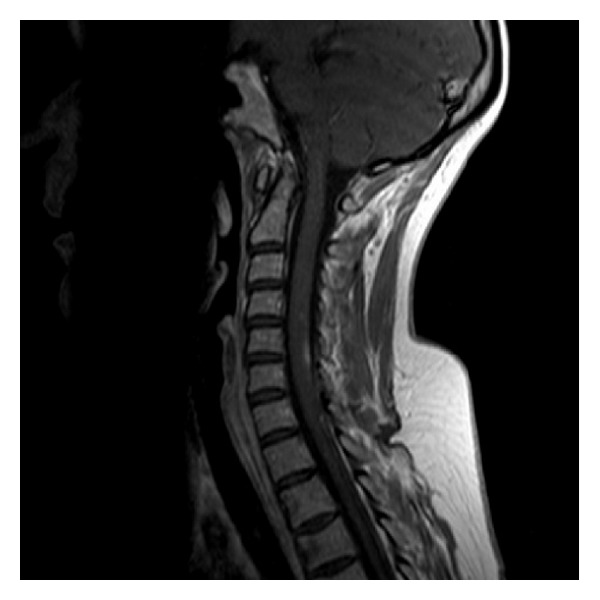
MRI spine showed a long intramedullary inflammatory lesion in the cord from C3 to the upper boarder of T1 on T1-weighted MRI imaging.

Investigations for neuropathy showed a folate level slightly reduced at 2.2 ng/mL and a normal Vitamin B12. She was seronegative for rheumatoid factor and negative for aquaporin-4 antibody (to exclude Devic's disease). Antinuclear antibody (Hep-2) was positive at 1 : 160 with a speckled pattern but anti-dsDNA antibody was negative making lupus less likely. Lumbar puncture biochemistry and microbiology were normal, with no oligoclonal band, and MRI head was also normal. MRI demonstrated abnormal signal return from the cervical spine from the level of C3 to the upper boarder of T1 (Figure 1). A diagnosis of cervical myelitis was made. Etanercept was discontinued four months after the development of neuropathy, with no noticeable improvement in her condition over the next six months. Despite the addition of folate supplements, a short course of methylprednisolone and amitriptyline, she continues to have significant neurological symptoms with impaired mobility and walking stamina.

## 2. Discussion

Inhibition of TNF, a cytokine critically involved in the acute inflammatory response in RA, is an established treatment for autoimmune arthropathy. Etanercept is a soluble (TNF-a) receptor-Fc fusion protein which specifically targets and inhibits the effects of TNF. Systematic review has shown it to be an effective treatment of RA with better disease control for treatment of inflammatory arthritides which are resistant to disease-modifying treatment and to provide an improved functional outcome [1]. Etanercept is recommended as a treatment option for patients who fail to respond adequately to at least two other disease-modifying drugs, in accordance with the British Society of Rheumatology (BSR) Guidelines [2].

Numerous studies have suggested a possible association between anti-TNF medications and demyelination. A double-blind placebo-controlled phase II study of 168 patients with relapsing-remitting multiple sclerosis (MS) showed that anti-TNF-treated patients experienced a significant number of exacerbations in comparison to controls [3]. Another study of 202 patients with inflammatory bowel disease receiving infliximab therapy showed 6 cases of suspected demyelination (three with confirmed neurological disease) [4]. A large case-control study of patients with RA showed that when controlling for differential prescription patterns, more demyelinating events occurred in patients after exposure to anti-TNF agents than RA patient controls [5]. Case reports suggest neurological side effects with anti-TNF therapy. Two patients have been described who developed a chronic inflammatory demyelinating polyneuropathy during their course of therapy with TNF-alpha antagonists [6]. Case series and reports show varying timing from initiation of treatment to onset of neurological symptoms from 6 to 21 months [7]. The proposed pathogeneses of TNF-alpha-associated neuropathies include both a T cell and humoral immune attack against peripheral nerve myelin, vasculitis-induced nerve ischemia, or inhibition of signalling support for axons [8].

A recent review article stresses that patients with RA are more at risk of developing neurological events in the future than non-RA patients, regardless of treatment with anti-TNF [7]. However, the authors did recommend that patients with a history of MS or MS-like illness are not good candidates for anti-TNF and that any patients who develop new or unusual neurological symptoms should stop anti-TNF treatment and have formal neurological assessment.

Although cervical myelitis appears to be a rare adverse effect of anti-TNF therapy, it is important that physicians are alert to the possible association of demyelination with treatment and adverse reactions are appropriately reported [8].
